# Supplementary material for: Arsenic hexoxide has differential effects on cell proliferation and genome-wide gene expression in human primary mammary epithelial and MCF7 cells
Source: Sci Rep. 2021 Feb 12;11:3761. doi: 10.1038/s41598-021-82551-3 (PMC7881197; doi:10.1038/s41598-021-82551-3)
Supplement: Supplementary file 1 — Supplementary Information. [file 41598_2021_82551_MOESM1_ESM.docx]

**SUPPLEMENTARY INFORMATION**

**––––––––––––––––––––––––––––––––––––––––––––––––––––––––––––––––––––––**

**Arsenic hexoxide has differential effects on cell proliferation and genome-wide gene expression in human primary mammary epithelial and MCF7 cells**

**Authors:** Donguk Kim^1^*, Na Yeon Park^2^*, Keunsoo Kang^3^, Stuart K. Calderwood^4^, Dong-Hyung Cho^2^, Ill Ju Bae^5^†, and Heeyoun Bunch^1^†

†Correspondence to: HB at [heeyounbunch@gmail.com](mailto:heeyounbunch@gmail.com)

**This PDF file includes:**

Supplementary Fig. 1–4

Supplementary Table 1

**SUPPLEMENTARY FIGURES**

**A.**

**Microscopic images at 24 hours**

**
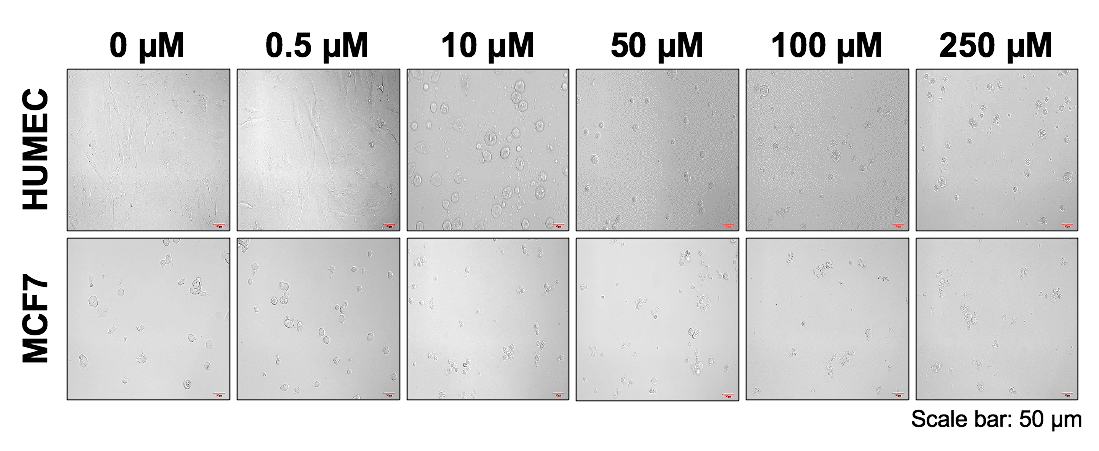
**

**B.**

**
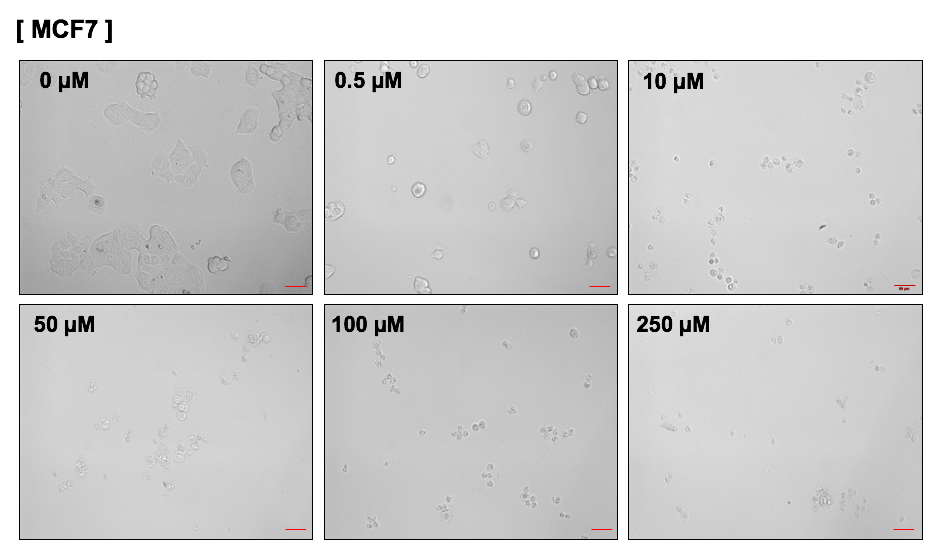

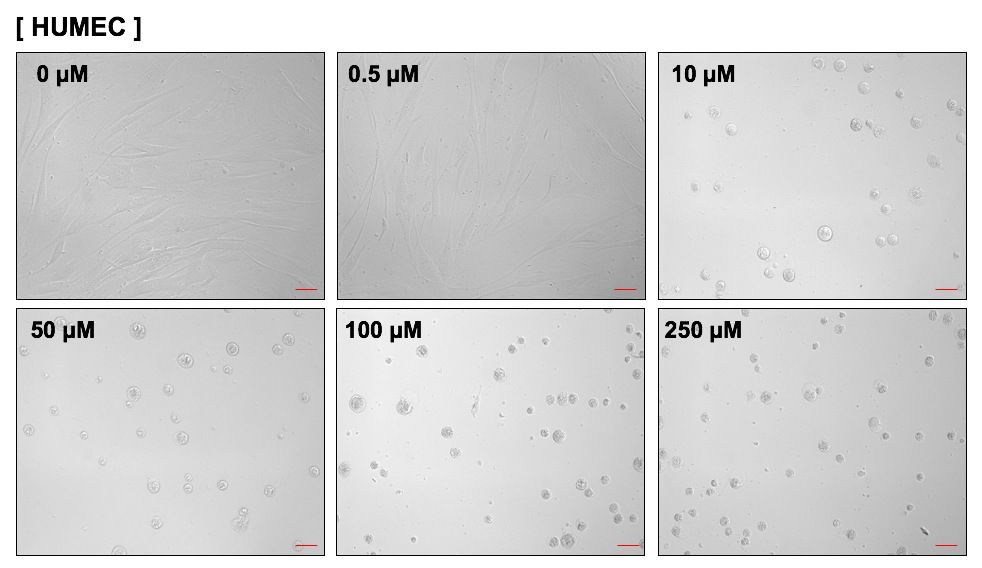
Microscopic images at 48 hours**

**C.**

**Microscopic images at 72 hours**

**
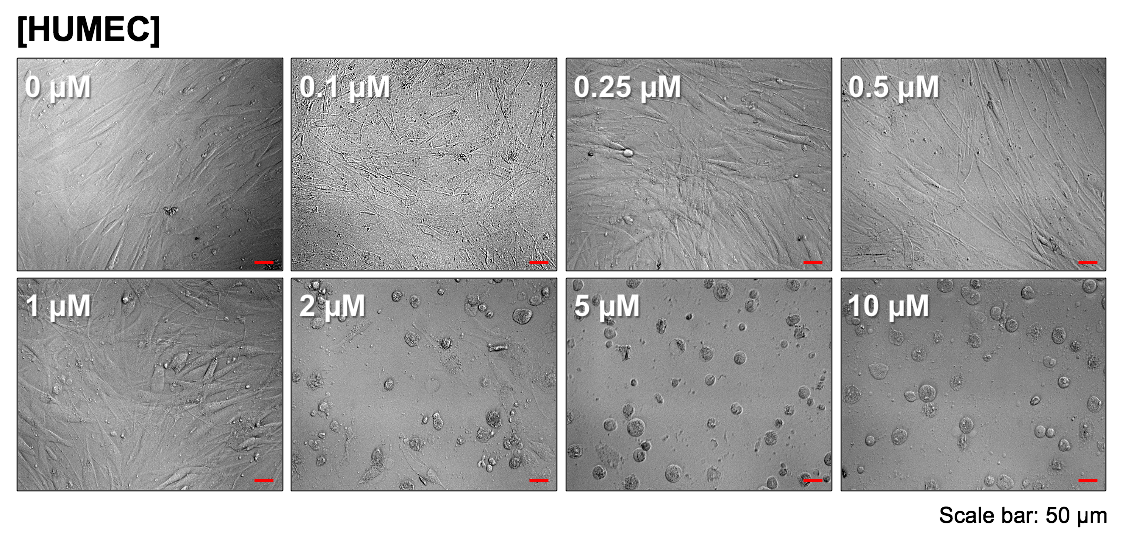
**

**
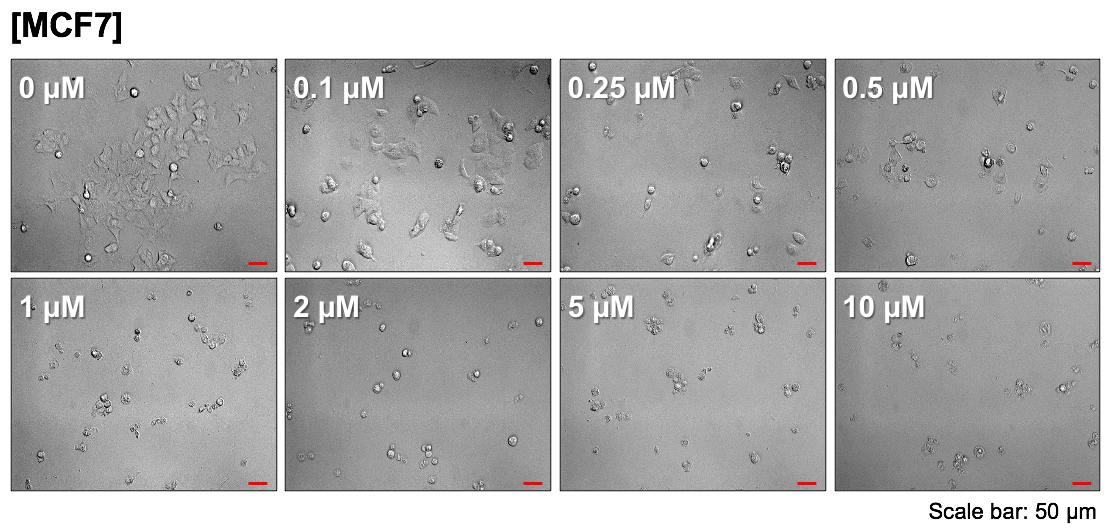
**

**Supplementary Figure 1**

Cytotoxicity analysis- time-course microscopic images of AS_4_O_6_ (AS6)-treated HUMEC and MCF7 cells

(A) Microscopic images at 24 h after cells were treated with AS6 at given concentrations. HMEC, HUMEC. (B) Microscopic images at 48 h after cells were treated with AS6 at given concentrations. (C) Microscopic images at 72 h after cells were treated with AS6 at given concentrations, showing sharp contrasts in cell viability between HUMEC and MCF7 cells at the concentrations below 1 μM. Scalebar = 50 μm.

**A.**

**HUMEC (all genes, n = 81702)**

**
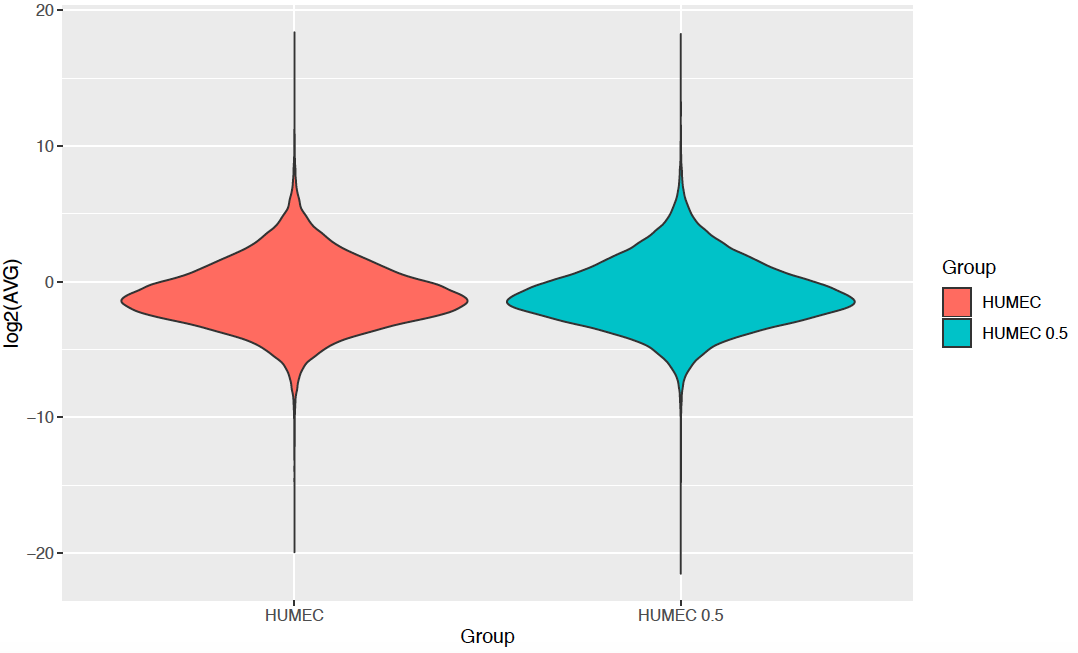
**

**MCF7 (all genes, n = 91144)**

**
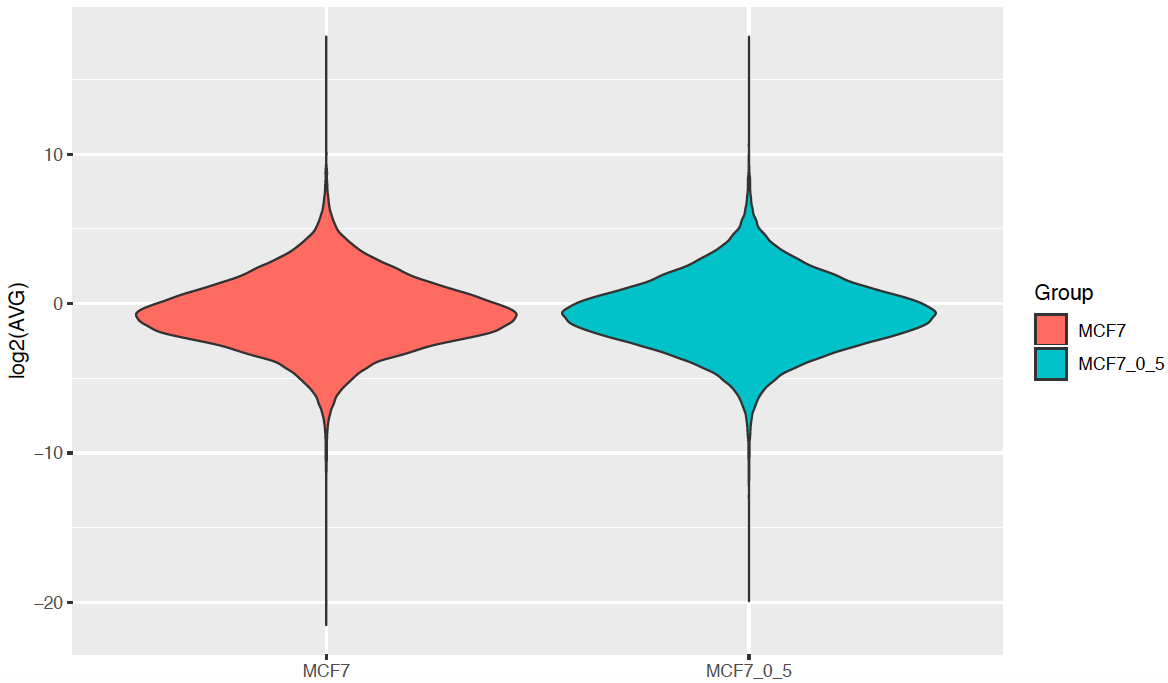
**

**Supplementary Figure 2**

RNA-seq and genomic analysis

(A) Violin plots showing overall transcriptome changes mediated by AS6 in HUMEC and MCF7 cells.

**A.**

**HUMEC up-regulated (PPI, n= 486 genes)**

**
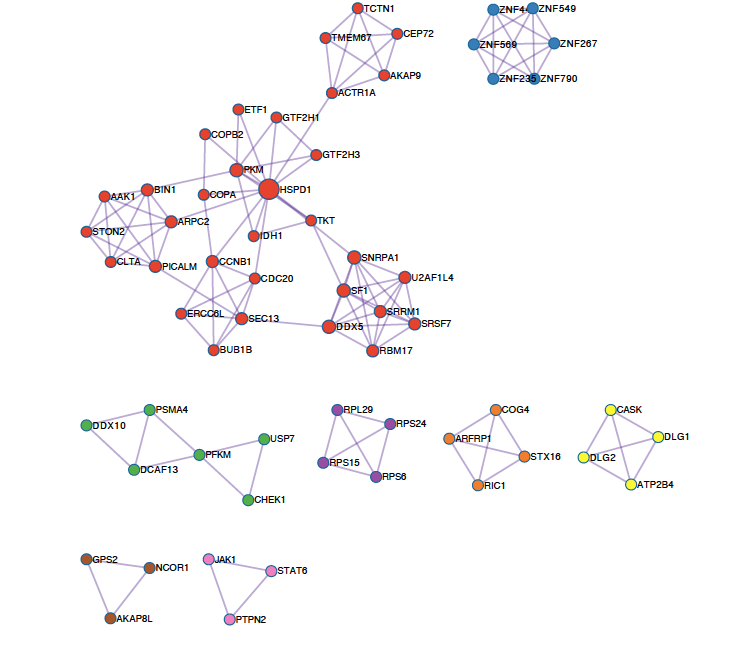
**

**B.**

**HUMEC down-regulated (PPI, n= 415 genes)**

**
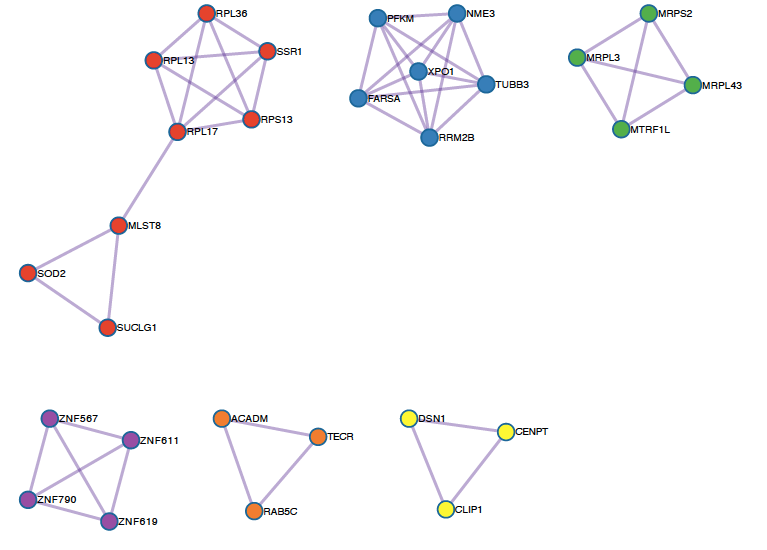
**

**C.**

**MCF7 up-regulated (PPI, n= 449 genes)**

**
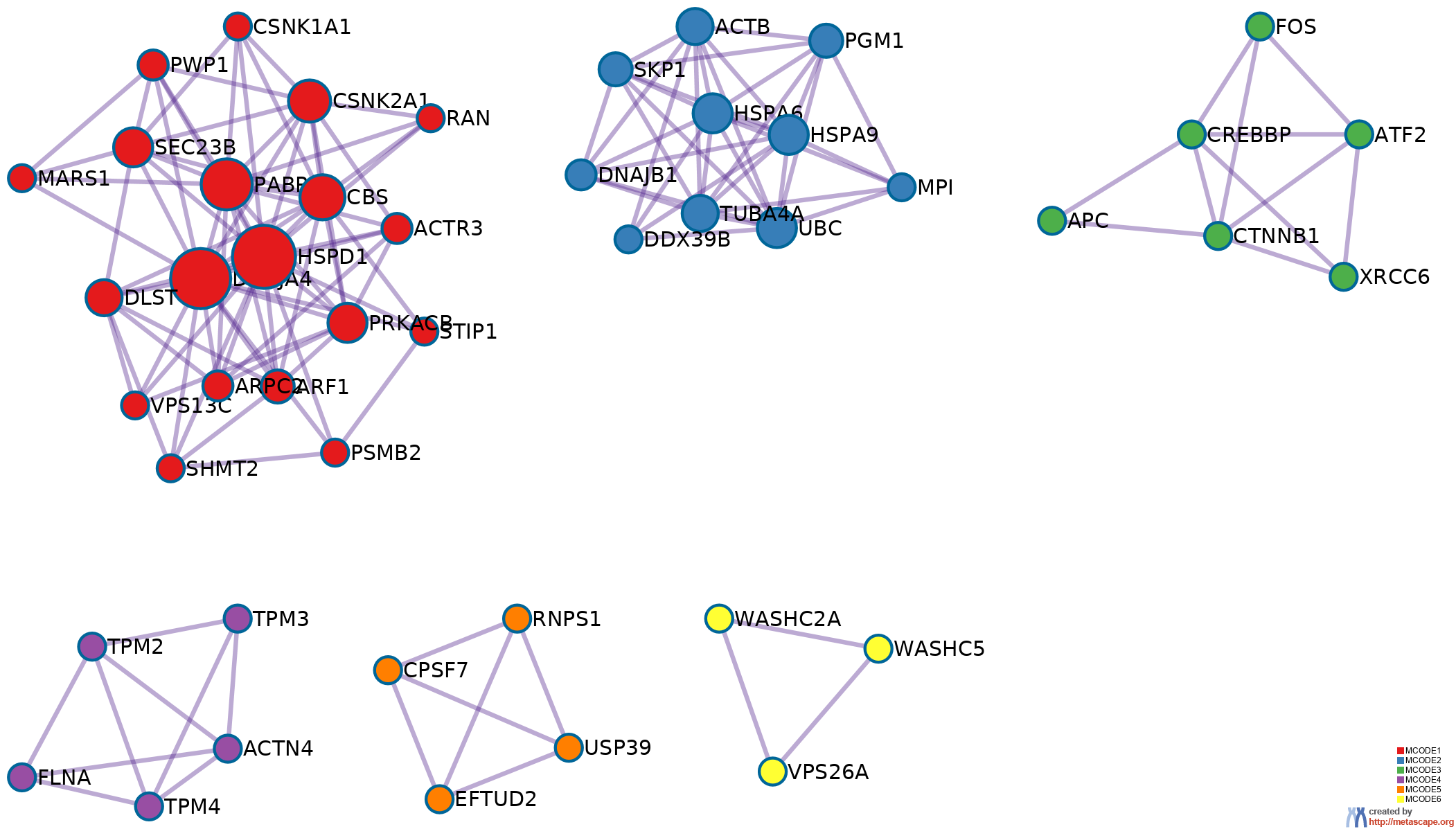
**

**D.**

**MCF7 down-regulated (PPI, n= 491 genes)**

**
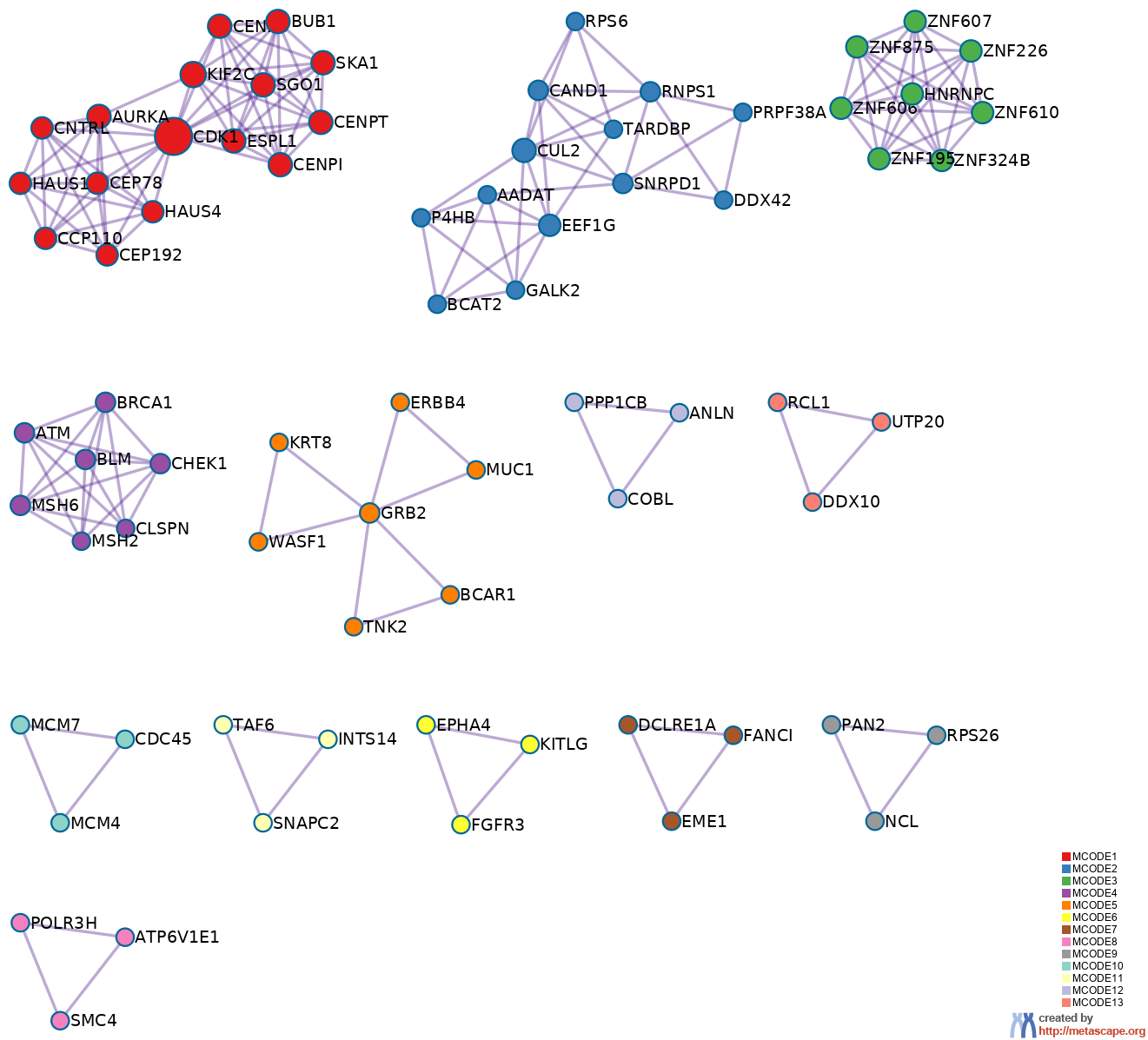
**

**Supplementary Figure 3**

Protein-protein interaction (PPI) network analysis of differentially expression genes by RNA-seq

(A) Upregulated genes upon AS6 treatment in HUMEC. (B) Downregulated genes upon AS6 treatment in HUMEC. (C) Upregulated genes upon AS6 treatment in MCF7 cells. (D) Downregulated genes upon AS6 treatment in MCF7 cells.

**A.**

**
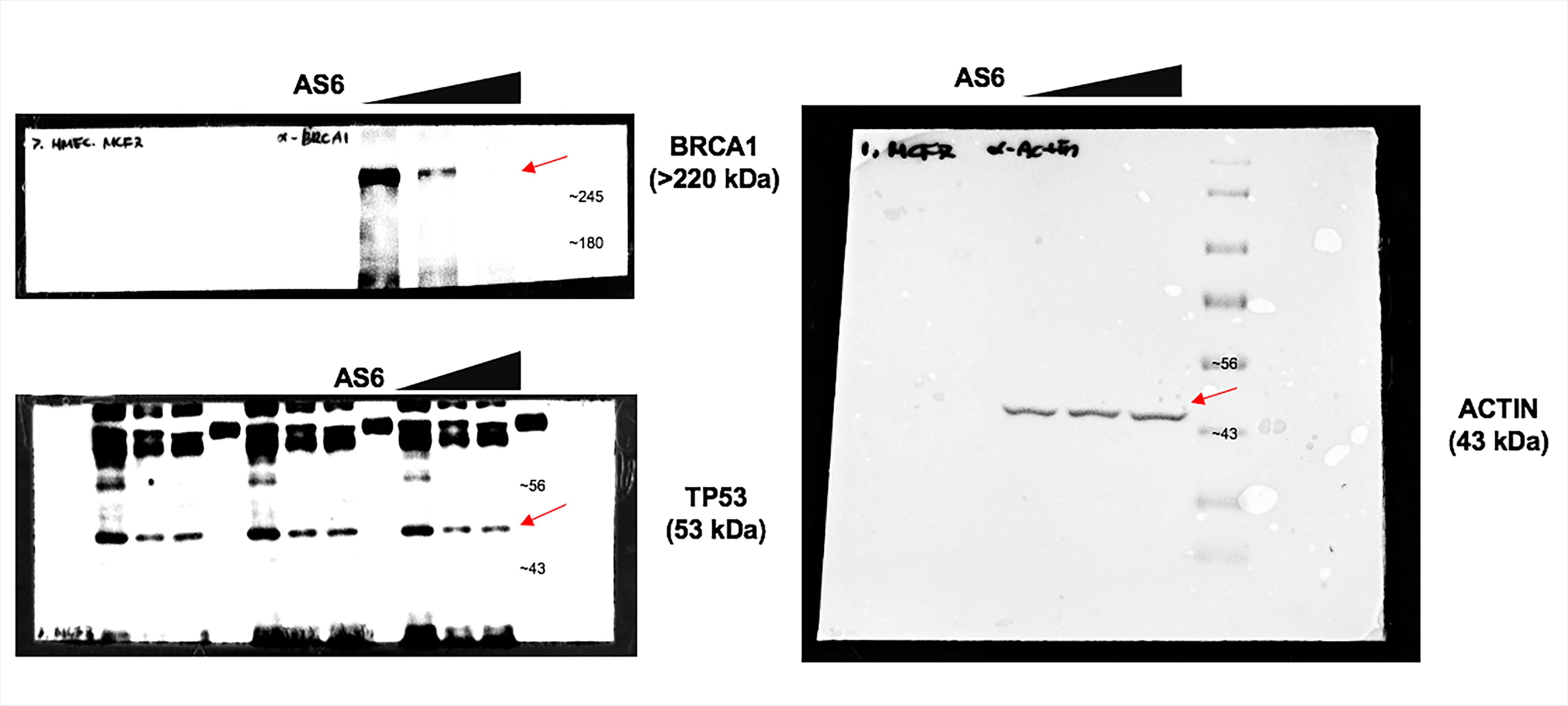
**

**B.**

**
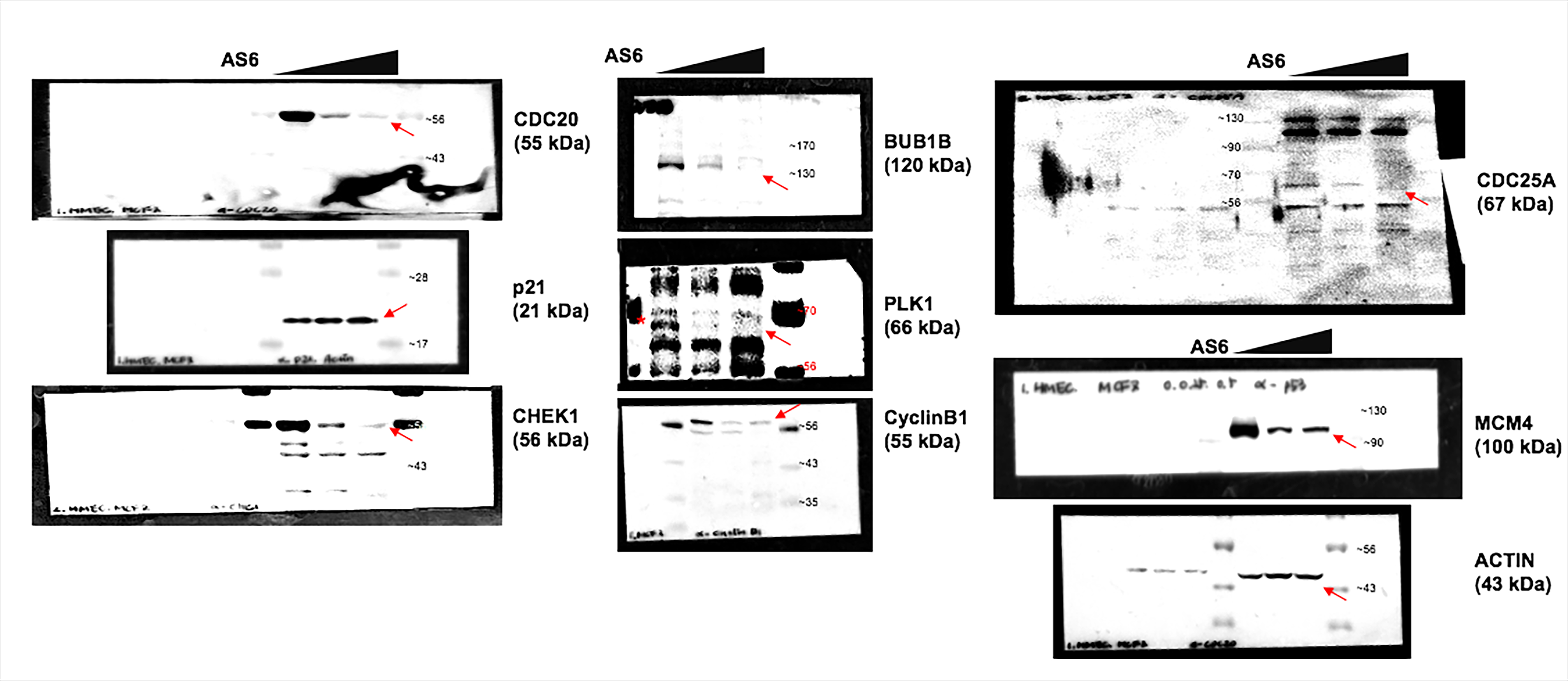
**

**C.**

**
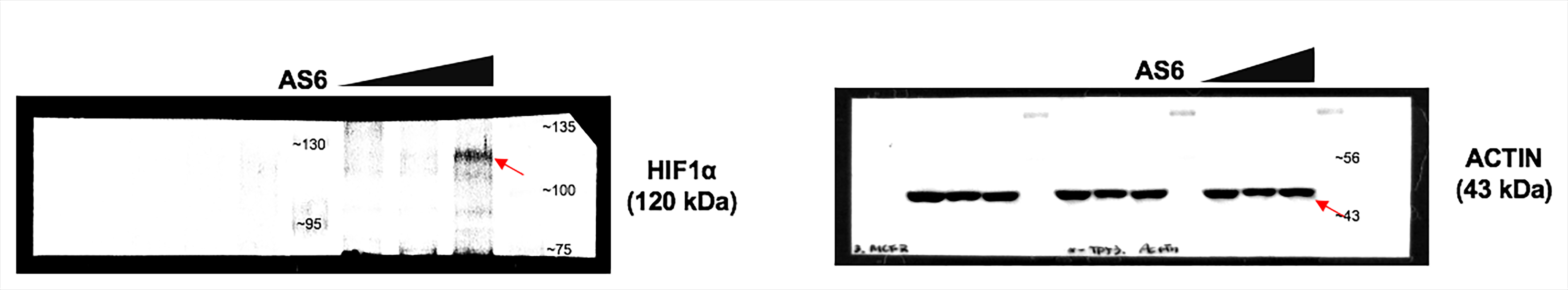
**

**D.**

**
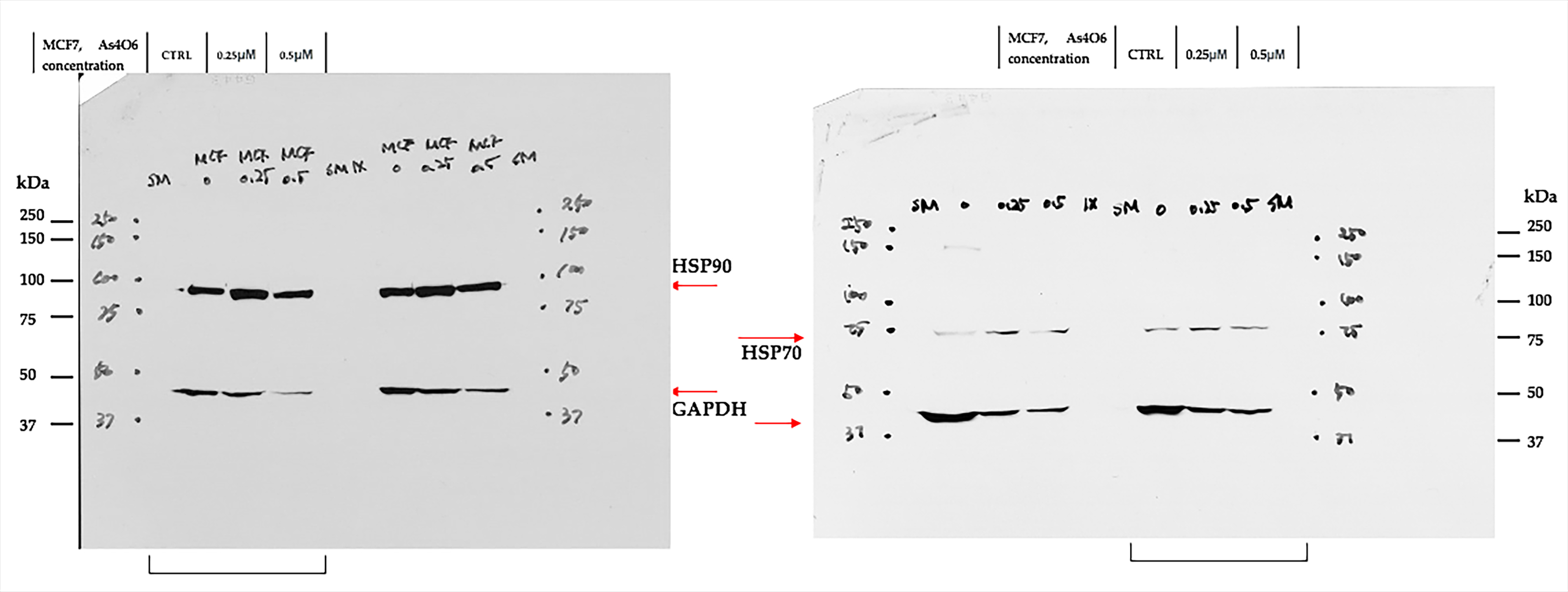
**

**Supplementary Figure 4**

Uncropped and full-length of blots in immunoblotting analyses

(A) DNA damage response proteins. (B) Cell cycle regulators. (C) HIF1α. (D) HSP70 and HSP90.

**SUPPLEMENTARY TABLES**

Supplementary Table 1.

The sequences of oligos and primers used in this study

| **For Real-time PCR** | |
| --- | --- |
| **CHEK1-Forward** | 5'- GGT GAA TAT AGT GCT GCT ATG TTG ACA -3' |
| **CHEK1-Reverse** | 5'- TTG GAT AAA CAG GGA AGT GAA CAC -3' |
| **CDC20-Forward** | 5'- GAC CAC TCC TAG CAA ACC TGG -3' |
| **CDC20-Reverse** | 5'- GGG CGT CTG GCT GTT TTC A -3' |
| **CDC25A-Forward** | 5'- CAG CTT CCA CAC CAG TCT CT -3' |
| **CDC25A-Reverse** | 5'- TTG ACT GCC GAT ACC CAT AT -3' |
| **MCM4-Forward** | 5'- TGT GGC AGC ATG CAA AGA A -3' |
| **MCM4-Reverse** | 5'- GGG TCA ATA AAA CGC TGA AGA AA -3' |
| **BUB1B-Forward** | 5'- ACG TTA TTA GAA AGA GCT GTA G -3' |
| **BUB1B-Reverse** | 5'- CAT ATC CAA AGG CTC ATT GC -3' |
| **p53-Forward** | 5'- ATG AGC CGC CTG AGG TTG -3' |
| **p53-Reverse** | 5'- AGC TGT TCC GGA GGC CCA -3' |
| **BRCA1-Forward** | 5'- GCA GAG AGT CAG ACC CTT CAA TGG -3' |
| **BRCA1-Reverse** | 5'- GCC CAG GTT TCA AGT TTC CTT TTC -3' |
| **ATM-Forward** | 5'- CAG GCG AAA AGA ATC TGG GG -3' |
| **ATM-Reverse** | 5'- GCA CAA AGT AGG GTG GGA AAG C -3' |
| **CDK1-Forward** | 5'- AAC TTT CGC CTG AGC CTA TT -3' |
| **CDK1-Reverse** | 5'- ATC AGC TCC ATC TTC TGC ATC C -3' |
| **HSP70-Forward** | 5'- ATG TCG GTG GTG GGC ATA GA -3' |
| **HSP70-Reverse** | 5'- CAC AGC GAC GTA GCA GCT CT -3' |
| **HSP90-Forward** | 5'- CCG TTT CTG AGA AGC AGG GCA -3' |
| **HSP90-Reverse** | 5'- CTG TCT GAA GGC CAG TGA CG -3' |
| **HIF1A-Forward** | 5'- GAA AGC GCA AGT CTT CAA AG -3' |
| **HIF1A-Reverse** | 5'- TGG GTA GGA GAT GGA GAT GC -3' |
| **CASP9-Forward** | 5'- ATG ACC ACC ACA AAG CAG TCC -3' |
| **CASP9-Reverse** | 5'- CGT GAC CAT TTT CTT GGC AG -3' |
| **ACTIN-Forward** | GCC GAC AGG ATG CAG AAG GAG ATC A |
| **ACTIN-Reverse** | AAG CAT TTG CGG TGG ACG ATG GA |
